# Correlation of the Hallux Sesamoids’ Orientation with Various Anatomical Parameters in Patients with Hallux Valgus Deformity

**DOI:** 10.7759/cureus.4643

**Published:** 2019-05-11

**Authors:** Kalliopi Iliou, George K Paraskevas, Panagiotis Kanavaros, Alexandra Barbouti, Aikaterini Kitsouli, Christos Gekas, Panagiotis Kitsoulis

**Affiliations:** 1 Psychiatry, Aristotle University of Thessaloniki, Thessaloniki, GRC; 2 Orthopaedics, Aristotle University of Thessaloniki, Thessaloniki, GRC; 3 Anatomy-Histology-Embryology, University of Ioannina, Ioannina, GRC; 4 Anatomy, Aristotle University of Thessaloniki, Thessaloniki, GRC; 5 Orthopaedics, University of Ioannina, Ioannina, GRC

**Keywords:** intermetatarsal angles, first metatarsal head, hallux valgus angle

## Abstract

Introduction

An awareness of the anatomical parameters of the foot such as the position and orientation of the sesamoid bones can be of great value for the etiology and diagnostic approach to patients with hallux valgus (HV). The purpose of this study was to evaluate the basic anatomical features and measurements related to the characteristics of HV in cadaveric material.

Materials and methods

The study sample included 12 cadaveric limbs with HV and 10 cadaveric limbs without HV as a control group. We measured the HV angle (HVA), the first to second intermetatarsal angle (IMA), and the first to fifth IMAs of all samples. We also recorded the shape of the first metatarsal head and the position/orientation of the sesamoid bones.

Results

The mean values of the HVA (p = 0.000), the first to second IMA (p = 0.000), and the first to fifth IMA (p = 0.000) differed between the HV and non-HV group. The position of the sesamoid ossicles between the HV and the non-HV group was statistically significant (p = 0.001). While we noted the round shape of the first metatarsal head was predominant in HV samples, we found no statistically significant difference in the first metatarsal head shape between the test and control groups.

Conclusion

The HVA, the first to second IMA, the first to fifth IMA, and the orientation of the sesamoid ossicles differed significantly between cadaveric samples with HV and those without HV.

## Introduction

Hallux valgus (HV) is a progressive forefoot deformity associated with foot pain, decreased functional status, cosmetic disturbance, and worsened foot health [1,2]. As the HV deformity increases, it becomes more painful and debilitating for patients [3]. HV occurs in up to 1% of the general adult population [4] and is more common in women and elderly patients [5]. In elderly women, HV has an incidence of up to 16% [6].

The diagnosis of HV is usually based on roentgenographic measurements. The HV angle (HVA) and the first to second intermetatarsal angle (IMA) are the basic roentgenographic measurements; healthy values are <15° HVA and <9° first to second IMA. Additional roentgenographic findings can often be of great value for the etiology and diagnostic approach, especially in preoperative planning for patients with HV. Some of these additional findings include the first metatarsal head shape and the sesamoid bones position [7].

We conducted this study to display the basic anatomical features and measurements related to characteristics of HV in cadaveric material. Our goal was to highlight the potential association of the hallux sesamoids’ orientation, the first metatarsal head’s shape, the HVA, first IMA, and first to fifth IMA on the appearance of the HV deformity.

## Materials and methods

The study sample included 12 cadaveric limbs with HV (11 women’s feet and one man’s foot) and 10 cadaveric limbs without HV deformity as a control group (nine women’s feet and one man’s foot). The cadaveric material was derived from the cadaveric collection of fresh-frozen feet of the Department of Anatomy-Histology-Embryology of the Medical School of the University of Ioannina. Using classical methods of human dissection, we resected the skin overlying the dorsal and plantar aspect of the foot. Furthermore, after meticulous preparation, we removed the extensor and flexor tendons of the great toe to demonstrate the sesamoid ossicles of the first metatarsophalangeal joint. Ultimately, we excised the articular capsule of the aforementioned joint to reveal the precise shape of the first metatarsal head.

**Figure 1:**
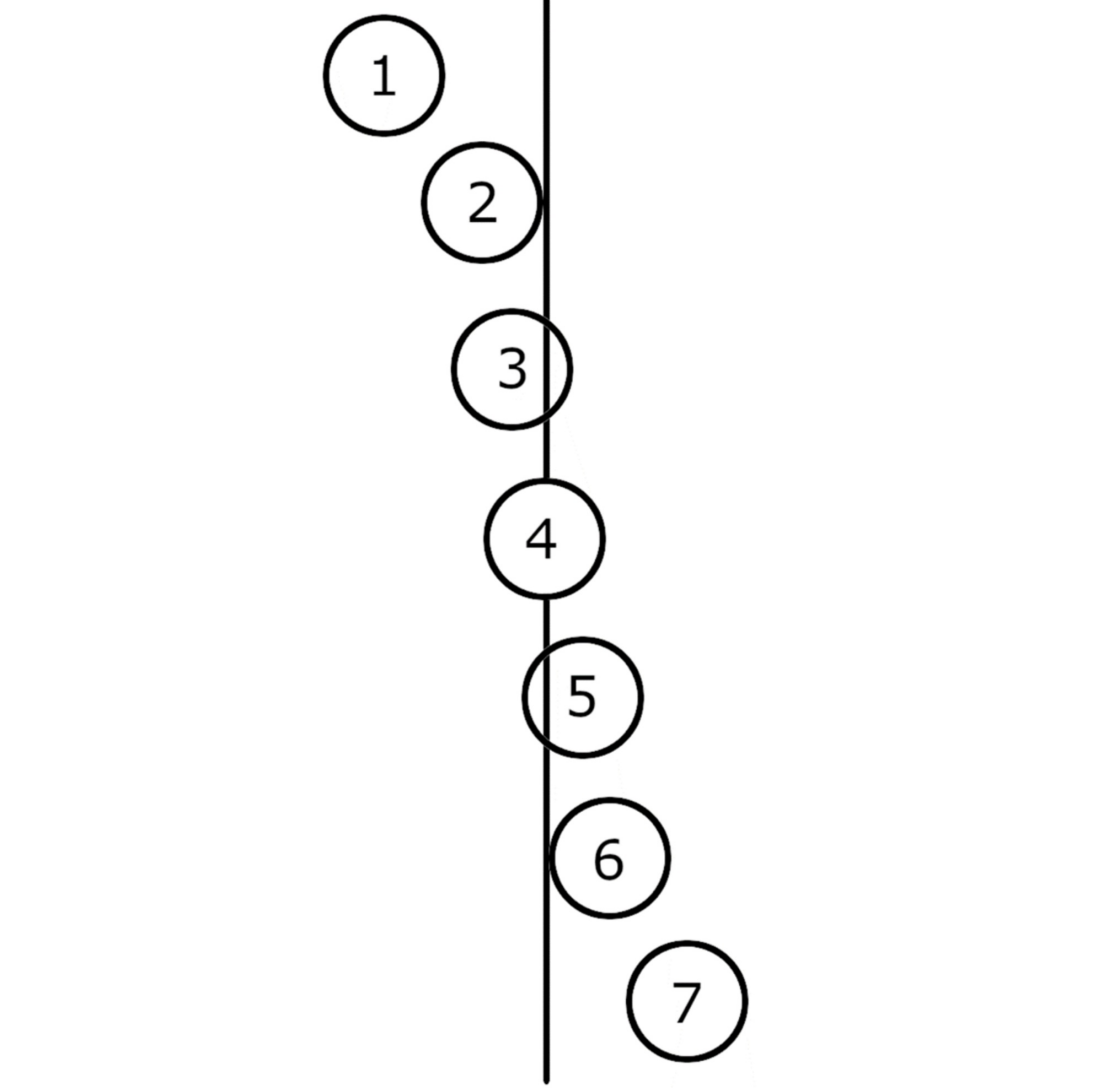
Different positions of the sesamoid ossicles of the first metatarsophalangeal joint with respect to the longitudinal axis of the first metatarsal bone. Schematic representation of the different positions of the sesamoid ossicles of the first metatarsophalangeal joint with respect to the longitudinal axis of the first metatarsal bone, as proposed by Hardy and Clapham [8].

We measured the HVA, first to second IMA, and the first to fifth IMA using a goniometer placed directly on the feet. The shape of the first metatarsal head and the position/orientation of the sesamoid bones were recorded. We detected two morphological patterns of the first metatarsal head (round and square). The position/orientation of the sesamoid ossicles with respect to the midline of the plantar surface of the first metatarsal head was recorded using the Hardy classification as a guide [8]. The first position was defined as the medial sesamoid located entirely medial to the long axis of the first metatarsal bone without being in contact with it. The second position was the medial sesamoid in contact with the axis. The third position was defined as <50% of the medial sesamoid surface crossed by the axis. The fourth position denoted the medial sesamoid divided into two isometric pieces by the axis, and the fifth position meant that >50% of the sesamoid surface was crossed by the axis. In the sixth position, the sesamoid was in contact with the axis. The seventh position meant the sesamoid bone was entirely lateral to the axis (Figure 1) [8].

All anatomical features were studied and recorded by two independent observers. We set up the interobserver and intraobserver error at p > 0.05. When p < 0.05, the relative detection was undertaken by a third independent observer.

The data were analyzed using PASW Statistics for Windows, Version 18.0. (SPSS, Inc., Chicago, IL). We used the Kolmogorov-Smirnov test and the Shapiro-Wilk test of normality to determine whether the data were normally distributed. For data analysis, we used Spearman’s rank correlation, chi-square test, and the nonparametric Mann-Whitney U test. The frequencies, the means and standard deviations of the variables were recorded for descriptive statistical analysis. A p-value <0.05 was considered statistically significant. The confidence interval was 95%.

## Results

As expected, the mean values of the HVA (p = 0.000) and the first to second IMA (p = 0.000) were statistically significantly higher than the corresponding values from the control group. The first to fifth IMA differed between the HV and non-HV group as well (p = 0.000) (Table 1).

**Table 1:** The mean values of the HVA, the IMA and the IMA in HV and non-HV group. The mean values of the hallux valgus angle (HVA), the first to second intermetatarsal angle (IMA) and the first to fifth intermetatarsal angle (IMA) along with the standard deviations (SD) in parentheses are demonstrated in hallux valgus (HV) and non-HV group.

		Angles		
		HVA	First to second IMA	First to fifth IMA
Groups	HV feet	19.42° (SD: 2.27)	10.5° (SD: 1.56)	31.75° (SD: 3.57)
	Non-HV feet	11.5° (SD: 2.22)	7.9° (SD: 0.99)	23.6° (SD: 2.91)

**Figure 2:**
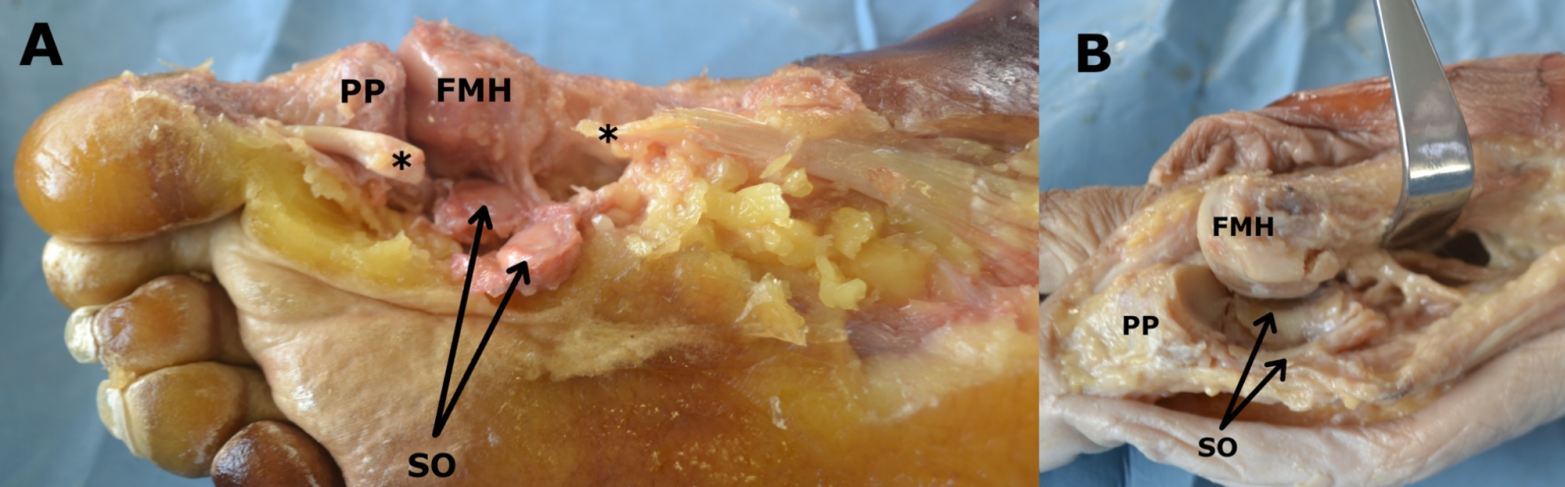
(A) The right first metatarsophalangeal joint is demonstrated after removal of its fibrous capsule, along with the sesamoid ossicles (SO) being reflected laterally. (B) The right first metatarsal head (FMH) has been disarticulated from the basis of the proximal phalanx (PP). (A) The right first metatarsophalangeal joint is demonstrated after removal of its fibrous capsule, along with the SO being reflected laterally. The cutting edges (asterisks) of the tendon of the long flexor muscle of the hallux are seen as well. (B) The right FMH has been disarticulated from the basis of the PP. The SO are demonstrated; the lateral one is seen articulated with the plantar articular aspect of the FMH.

There was a statistically significant difference between positions of the sesamoid ossicles in the HV and non-HV group (p = 0.001). The position of the sesamoids in the HV group was in the third to fifth IMA (Figure 2).

We found no case of bipartite medial or lateral sesamoid ossicles. While the round shape of the first metatarsal head was more common to the HV group, we found no statistically significant difference in the first metatarsal head shape between the groups.

## Discussion

The hallux sesamoid ossicles are located within the tendon of the flexor hallucis brevis and in a portion of the plantar plate. The abductor hallucis and adductor hallucis tendons have fibrous insertions into the medial and lateral sesamoids, respectively [9]. In plain radiographs, the sesamoid’s position can be evaluated according to a well-known scale that places the sesamoid bones in seven positions relative to the first metatarsal axis [8]. In normal positioning of the sesamoids, in positions one to three, the medial sesamoid articulates with the first metatarsal head crista. This contributes to the first metatarsophalangeal articulation stability. When the medial sesamoid is placed lateral to the metatarsal head crista, in positions four to seven, the first metatarsophalangeal articulation becomes unstable, and the hallux is further abducted [10]. In a study conducted by Okuda et al. in 65 patients with HV, the sesamoids were in the fifth to seventh positions in all the participants [11]. This finding is important because the migration of the sesamoids lateral to the axis of the first metatarsal bone places them in a stress position away from the first metatarsal. The presence of a bipartite sesamoid ossicle has been correlated with HV. The frequency of this finding is up to 14.6% [12,13]. We did not document bipartite sesamoid presence in the cadaveric material. The sesamoid ossicles’ positioning was found to be in the third to fifth positions in the HV sample feet and in the first and second positions in the control feet. In our material, the mean value of the HV angle and first to second IMA angle in all HV feet correspond to a mild HV deformity, which may explain the absence of extreme sesamoid positioning in the sixth and seventh positions.

The relationship between the shape of the first metatarsal head and the presence of HV remains controversial. According to Mann and Coughlin, the first metatarsal head can be round or square [14]. At the same time, the existence of a third shape for the first metatarsal, square with a central ridge, has been described [15,16]. The round shape of the first metatarsal head seems to be an important factor in HV development [17-19]. A square-shaped first metatarsal head seems to resist the deformation forces that lead to HV, but the shape is a predisposing factor for hallux rigidus development [20]. Okuda et al. focus on the shape of the lateral edge of the first metatarsal head and denote three shapes: round (R-type), angular (A-type), and intermediate (I-type) type [21]. They defined the round sign as positive when the shape of the lateral edge was classified as type R and negative when the lateral edge was classified as angular or intermediate. The prevalence of the R-type in this study was statistically significantly higher in the HV group compared to the control group. At the same time, the round-shaped head in postoperative plain radiographs was considered a predisposition factor for postoperative recurrence [21]. The positive round sign was recently associated with increased pronation and a decreased inclination of the first metatarsal head, and a negative round sign may be used as an indicator of correction efficacy of the first metatarsal pronation during HV surgery [22]. Kilmartin and Wallace contradict this statement on the influence of the round metatarsal head shape [23]. In their study of 100 juvenile first metatarsal heads in HV feet, they observed only 40 samples with a round shape. They concluded the assessment of the first metatarsal head shape does not influence the potential development of first metatarsophalangeal joint pathology [23]. In our study, we found no statistically significant difference in shape between the first metatarsal head shape of the HV and the control groups, which aligns with the report by Kilmartin and Wallace.

The investigations regarding the first to fifth IMA in the literature are relatively rare. The normal values of this angle are between 14° to 35° [24]. The foot width can be evaluated using this angle measurement, especially in pathological conditions such as the bunionette deformity (Taylor’s bunion) [25,26]. Price stated that the first to fifth IMA is a more reliable indicator of first metatarsal varus deformity than the first to second IMA in patients with HV [27]. In a more recent study concerning the changes in the radiographic appearance during weight-bearing and non-weight-bearing in patients with HV, Gong et al. claimed that there was an increase of the angles’ values on weight-bearing X-rays, and there was a moderate relationship between the changes noted in the first to second IMA, the first to fifth IMA, and the degree of the HV deformity. He concluded that the pain under the first metatarsal head of HV is influenced positively by the first to second and first to fifth IMAs [28]. In our study, we found a statistically significant difference between the groups of HV feet and normal feet regarding the first to fifth IMA, but, at the same time, the mean value of that angle was normal in both groups. Such a finding suggests that the foot width is increased in a patient with HV. However, the assessment of first to fifth IMA does not constitute a reliable and useful tool for the determination of the degree of HV deformity.

## Conclusions

The HVA, the first to second IMA, the first to fifth IMA, and the orientation of the sesamoid ossicles differed between cadaveric feet with HV and those without HV. While the round shape of the first metatarsal head was more common in the HV group, there was no statistically significant difference of the first metatarsal head shape between the HV and control groups.
